# Transcription Factors Responding to Pb Stress in Maize

**DOI:** 10.3390/genes8090231

**Published:** 2017-09-18

**Authors:** Yanling Zhang, Fei Ge, Fengxia Hou, Wenting Sun, Qi Zheng, Xiaoxiang Zhang, Langlang Ma, Jun Fu, Xiujing He, Huanwei Peng, Guangtang Pan, Yaou Shen

**Affiliations:** 1 Key Laboratory of Biology and Genetic Improvement of Maize in Southwest Region, Maize Research Institute, Sichuan Agricultural University, Chengdu 611130, China; jocelyn0315@126.com (Y.Z.); gefei511@163.com (F.G.); bingyimuhun@163.com (F.H.); m15102858130@163.com (W.S.); zhengqi345@126.com (Q.Z.); kfzhangxiaoxiang@163.com (X.Z.); sxyljxml@163.com (L.M.); 18280182459@163.com (J.F.); xiujinghe77@gmail.com (X.H.); pangt@sicau.edu.cn (G.P.); 2 Animal Nutrition Research Institute, Sichuan Agricultural University, Chengdu 611130, China; phw@sicau.edu.cn

**Keywords:** transcription factor, maize, stress, validation

## Abstract

Pb can damage the physiological function of human organs by entering the human body via food-chain enrichment. Revealing the mechanisms of maize tolerance to Pb is critical for preventing this. In this study, a Pb-tolerant maize inbred line, 178, was used to analyse transcription factors (TFs) expressed under Pb stress based on RNA sequencing data. A total of 464 genes expressed in control check (CK) or Pb treatment samples were annotated as TFs. Among them, 262 differentially expressed transcription factors (DETs) were identified that responded to Pb treatment. Furthermore, the DETs were classified into 4 classes according to their expression patterns, and 17, 12 and 2 DETs were significantly annotated to plant hormone signal transduction, basal transcription factors and base excision repair, respectively. Seventeen DETs were found to participate in the plant hormone signal transduction pathway, where basic leucine zippers (bZIPs) were the most significantly enriched TFs, with 12 members involved. We further obtained 5 *Arabidopsis* transfer DNA (T-DNA) mutants for 6 of the maize bZIPs, among which the mutants *atbzip20* and *atbzip47*, representing *ZmbZIP54* and *ZmbZIP107*, showed obviously inhibited growth of roots and above-ground parts, compared with wild type. Five highly Pb-tolerant and 5 highly Pb-sensitive in maize lines were subjected to DNA polymorphism and expression level analysis of *ZmbZIP54* and *ZmbZIP107*. The results suggested that differences in bZIPs expression partially accounted for the differences in Pb-tolerance among the maize lines. Our results contribute to the understanding of the molecular regulation mechanisms of TFs in maize under Pb stress.

## 1. Introduction

As a widely grown food crop, maize is important in supporting the growing world population. Maize production is threatened by lead pollution of soil, which results from atmospheric Pb deposition, sewage irrigation, waste accumulation, and metal mine acid waste water. Pb accumulates in roots, stems and other organs, and seriously affects the growth and development of plants [1]. More seriously, Pb can easily flow into animal and human bodies through the food chain and endanger their health [2]. 

Transcription factors (TFs) are a group of proteins that can specifically bind to 5′ upstream DNA regions and ensure target gene expression at a certain time and space; they are vital for the normal development of an organism, as well as for routine cellular functions [3,4]. Some TFs interact with *cis*-elements in the promoter regions of several stress-related genes and thus up-regulate the expression of many downstream genes, resulting in the imparting of abiotic stress tolerance [5]. TFs *AtbHLH17* and *TtWRKY28* are up-regulated under drought and oxidative stress, respectively, in *Arabidopsis*. Over-expression of these TFs up-regulates downstream target genes and improves abiotic stress tolerance [6]. The overexpression of Dehydration-Responsive Element-Binding Protein 2 (EsDREB2B), which exhibits transactivation activity of a GAL4-containing reporter, increases the tolerance to multiple abiotic stress factors including drought, salinity, cold, heat, heavy metals, and mechanical wounds in yeast [7]. *TabZIP60* enhances the resistance of multiple abiotic stress factors through the abscisic acid signalling pathway in wheat [8], and *Arabidopsis* constitutively overexpressing *BnbZIP3* is more tolerant to salinity and heavy metal (Cd) stress [9]. Moreover, overexpression of *ZmbZIP72* enhances the drought and other abiotic stress tolerances of transgenic *Arabidopsis* plants [10]. However, little is known about the regulation of transcription factors at the genome-wide level.

In this study, based on RNA sequencing data, we performed an expression analysis of transcription factors under Pb stress in a Pb-tolerant maize inbred line, 178. In total, we identified 464 transcription factors belonging to 50 families expressed under Pb stress. There were 262 transcription factors expressed differentially in three stages of Pb stress compared with the control solution (CK). Among these transcription factors, 11 could not be categorized into any known families. We further validated the functions of *ZmbZIP54* and *ZmbZIP107* on Pb tolerance through plant hormone signal transduction in maize. 

## 2. Materials and Methods

### 2.1. Samples and RNA Isolation

Inbred line 178 is a maize variety tolerant to relatively high concentrations of Pb [11], rendering it an excellent resource for discovery of novel factors conferring Pb tolerance. In this study, based on RNA sequencing data, we performed an expression analysis of transcription factors in the process of Pb stress in inbred line 178. Preparation of the samples and RNA isolation were both carried out as described by Shen et al. [12]. In short, seven-day seedlings of the uniform-growth maize inbred line 178 were randomly divided into two groups; one was transferred to fresh nutrient solution as control check (CK), and the other was transplanted into an identical solution, but containing an additional 3 mM Pb(NO_3_)_2_ (Pb treatment). The composition of the nutrient solution is shown in Table S1. TRIzol reagent (Thermo Fisher Scientific, Waltham, MA, USA) was used to extract total RNA from immature roots that were collected from samples at 0 h (CK), 24 h (stage I), 48 h (stage II), and 72 h (stage III) after Pb treatment separately and the samples at 0 h (CK), 24 h (CK), 48 h (CK), and 72 h (CK). The first four RNA samples were submitted to RNA-seq analysis and all seven samples were submitted to qRT-PCR analysis. 

### 2.2. Gene Annotation, Differentially Expressed TFs, Enrichment Analysis and Cluster Analysis

We screened for transcription factors in the available genome expression profile data of immature maize roots under Pb stress. Gene Ontology (GO) and pathway annotation was assigned to differentially expressed genes using web-based tools described by Ashburner et al. [13]. The genes mapped in the maize genome were annotated by the euKaryotic Orthologous Groups (KOG) functional classification [14]. Compared with CK, the genes whose expression in any of the three stages changed by at least two-fold (log_2_ ratio > 1 or < −1) with a *p*-value < 0.05 were used for further analysis. The differentially expressed TFs were then clustered according to their expression patterns by using the software Express Cluster (npm, Oakland, CA, USA). To test the statistical enrichment of differentially expressed transcription factors (DETs) in Kyoto Encyclopedia of Genes and Genomes (KEGG) pathways, KOBAS software [15] was used for pathway enrichment analysis.

### 2.3. Real-Time PCR

Fourteen differentially expressed TF genes (GRMZM2G117961, GRMZM2G001930, GRMZM2G153333, GRMZM2G147712, GRMZM2G060290, GRMZM2G368909, GRMZM2G113127, GRMZM2G069370, GRMZM2G052102, GRMZM2G089361, GRMZM2G154641, GRMZM2G019446, GRMZM2G151826, and GRMZM2G169270) were randomly selected for quantitative real-time PCR analysis (ABI 7500 real-time PCR System, Torrance, CA, USA) to validate the differentially expressed genes (DEGs) obtained from Solexa sequencing. *Actin 1* (GRMZM2G126010) was used as the reference gene. The primers (Table S2) were designed using the software Primer Premier 5. Seven RNA samples were submitted to reverse transcription using a PrimeScript® RT reagent kit with gDNA Eraser (TaKaRa, Beijing, China). The PCR amplification programmes were performed in triplicate as described by Shen et al. [16]. Briefly, a 20-μL PCR reaction contained approximately 100 ng of cDNA, 10 μL of SYBR solution, and 200 nM primers. The 2^−∆∆Ct^ method was used for statistical analysis. The same method was applied to identify expression level diversity of *ZmbZIP54* and *ZmbZIP107* among various maize inbred lines, and the primers are shown in Table S2. 

### 2.4. Identification of *Arabidopsis* Mutant Pb Tolerance

To address the role of maize bZIPs in Pb tolerance, we performed phenotypic analysis of several T-DNA knockout alleles in *Arabidopsis*. The wild-type columbia *Arabidopsis* (ABRC, Columbus, OH, USA) and mutant seeds were surface-sterilized and vernalized at 4 °C for 3 days, then germinated on Murashige-Skoog (MS) medium (0.8% agar) for 1 week under a 16/8 h light/dark photoperiod at 23 °C and then transferred to MS media supplemented with either 0 mg/L or 200 mg/L Pb(NO_3_)_2_. Plant survival was monitored after one week of Pb (NO_3_)_2_ treatment. After seven days, the growth of the roots and above-ground parts were investigated for the mutants and the wild type (WT).

### 2.5. Intracellular Localization of Transcription Factors

Intracellular localization of TFs was carried out by the instantaneous expression of the TF–green fluorescence protein (GFP) fusion proteins in *Oryza* protoplasts. The isolation of protoplasts from rice and the subcellular localization assays were performed as described by Zhang et al. [17]. The TFs, *ZmbZIP54* (cloned by the 5′ primer: *GGGTACCCGG*GGATCCATGGCAGATGCTAGCTCAAGG, BamHI site underlined; 3′ primer: *CGACTCTAGA*GGATCCTTCACGTGGCCTAGCAAGCCAC, BamHI site underlined) and *ZmbZIP107* (cloned by the 5′ primer: *GGGTACCCGG*GGATCCATGGAGCTTTACCCTGGATATC; 3′ primer: *CGACTCTAGA*GGATCCTGCGGGCTCCCTGGGGCGCGCCG) were constructed into the pCAMBIA2300-35s-eGFP vector (Figure S1) [18]. The resulting fragments were separately cloned to the downstream of the constitutive cauliflower mosaic virus (*CaMV*) 35S promoter by the BamHI site of the pCAMBIA2300-35s-eGFP vector containing the GFP reporter gene.

## 3. Results and Discussion

### 3.1. The TFs Regulated by Pb: A Summary

**Figure 1:**
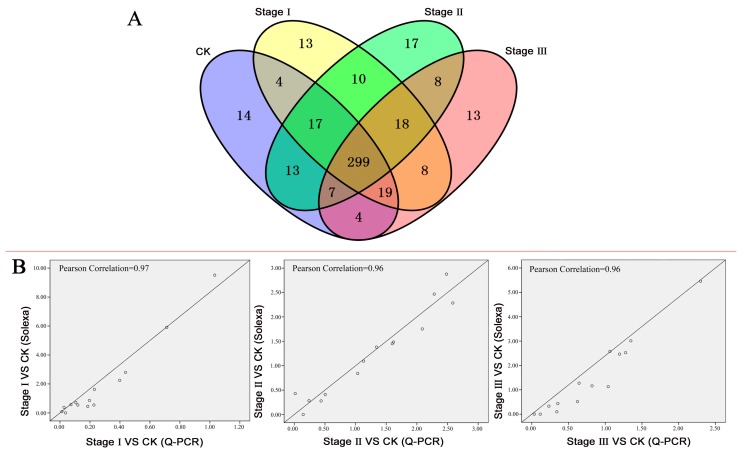
Transcription factor identification and quantitative Polymerase Chain Reaction (qPCR) validation. (**A**) Venn diagram showing the numbers of TFs expressed in control check (CK), stage I, stage II and stage III. Each oval indicates the numbers of transcription factors (TFs) in CK or each treatment. (**B**) Correlations of the expression ratios of TFs between qPCR and deep sequencing. The x-axis and y-axis represent the expression ratios of the stage sample to CK by qPCR and deep sequencing. The Pearson correlations in stage I VS CK, stage II VS CK, and stage III VS CK are 0.97, 0.96 and 0.96, respectively.

Based on KOG, GO and pathway analyses, we sought to identify the TF genes involved in responding to the heavy metal Pb. As a result, a total of 464 genes (1.99%) expressed in CK or Pb treatment samples were annotated as TFs from the RNA-seq. They were classed into 50 TF families according to their DNA-binding domains. The bZIP, GRAS, MADS-box, K-box, homeodomain-TF and CBF/NF-Y families were the largest, in which 99, 47, 40, 38, 31 and 28 members were expressed in at least one of the four libraries, respectively, followed by TCP, jumonji, SBP-box, Pathogenesis, TFIIB and WRKY. The proportion of these 12 families was 76.98% (Figure S2). The results showed that 377, 388, 389 and 376 TFs were expressed in the CK and stage I, II and III samples, respectively (Figure 1A). Of the 464 TFs, 299 (64.44%) were expressed in each of the four libraries. In addition, 14 (3.02%) TFs were specifically expressed in CK; however, 87 (18.75%) TFs were not found in CK but were expressed specifically in stage I, II or III samples (Figure 1A). These TFs may be involved in the response to Pb stress, and may play important roles in tolerance to Pb stress by repressing or activating their downstream targets. Of these candidate transcription factors, 18 were found in all three stages, and 13, 17, and 13 TFs were specifically expressed in stage I, II and III, respectively (Figure 1A). 

### 3.2. Differentially Expressed TFs (DETs) and TF Families

**Figure 2:**
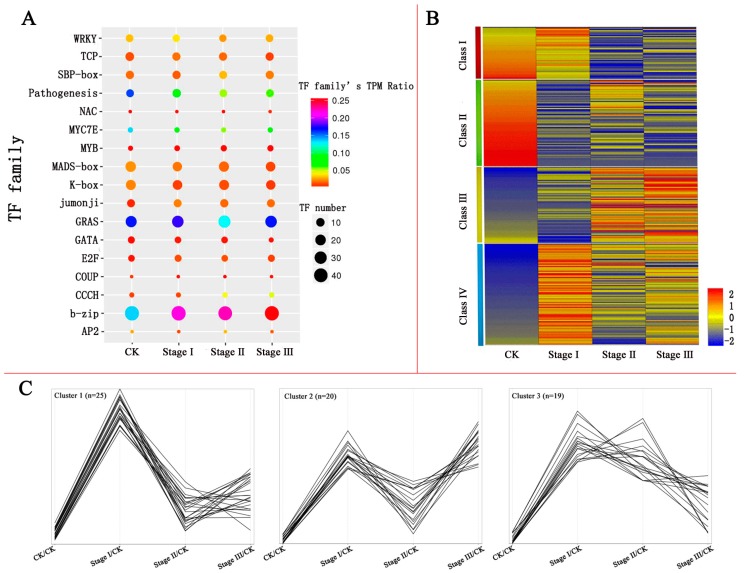
Frequencies of different classes of TF families and differentially expressed transcription factors (DETs) expression patterns in the process of Pb stress. (**A**) The 17 transcription factor families that are the most differentially expressed in the process of Pb stress. Solid circles represent the number of TFs involved in Pb stress. Different colours represent the TF family’s transcripts per million (TPM) ratio of the total TPM of all the DETs expressed in the family to the TPM of all TFs in the process of Pb stress. (**B**) Classes of all DETs involved in the response to Pb. (**C**) Clusters of DETs in Class IV may play important roles in resistance to Pb stress.

A total of 262 (56.5%) TFs in Pb treatment samples were identified as differentially expressed TFs (DETs) compared with CK (*p*-value < 0.05, and log_2_ fold change < −1 or > 1). Counts of 230, 231, and 227 DETs were identified for stage I, stage II, and stage III, respectively (Table S3). Among the DETs, approximately 77 (29.39%) were differentially expressed with more than five-fold change in any one of the stages. As the result of the analysis, 35 DETs were up- or down-regulated more than six-fold in the process of Pb treatment (Table 1). The 262 TFs were annotated into 43 families according to their DNA binding-domains (Table S4). Among them, the bZIP (51 members), GRAS (30 members), MADS-box (22 members), K-box (22 members), TCP (12 members), and SBP-box (11 members) families were the largest involved in Pb stress (Figure 2A). Compared with a previous finding that approximately 62.9% of TFs in immature maize embryos respond to exogenous 2, 4-D, and that MYB and BHLH are the largest families of DETs [19], our current research revealed that a smaller proportion of TFs and a different composition of TF families in maize roots participated in the response to Pb stress. 

### 3.3. Validation of the DET Expression Profiles by Quantitative RT-PCR

To verify the change in expression of the TFs based on deep sequencing, fourteen genes were validated by quantitative real-time PCR (qRT-PCR). The expression changes of these genes at 0 h (CK) and Pb treatment stages were consistent with the results of RNA-seq (Figure 1B). The results indicate a good agreement between deep sequencing and qRT-PCR methods. Since no CK condition was set for 24, 48, and 72 h treatments in RNA-seq experiments, we subsequently conducted qRT-PCR for the 14 genes for 0 h (CK), 24 h (CK), 48 h (CK), and 72 h (CK) samples in order to test whether developmental changes influence their transcriptional levels. Results showed no significant difference of TF expressions between each of the developmental stages and 0 h samples. Specifically, log_2_ developmental stage/0 h ranged from −1 to 1, and *p*-values were all larger than 0.05 except GRMZM2G368909 whose *p*-value was smaller than 0.05 (Table S5). This suggests that the identified DETs were mainly due to the response to Pb treatment instead of developmental changes.

### 3.4. Clustering of DETs Based on Their Expression Patterns after Pb Treatment

To explore significant differences in gene expression profiles between CK and stage I, stage II, and stage III, the co-modulated DETs were analysed using the K-means clustering algorithm. The 262 DETs were categorized into 4 distinct clusters (Figure 2B, Table S6). Class I contained 43 genes that were slightly changed in stage I but sharply down-regulated in stage II, with the lowest levels in stage III. A total of 72 genes in class II were sharply down-regulated in stage I, but they slightly rebounded in stage II and quickly returned to the lowest level in stage III. The expression patterns in class III genes (63 members) were the opposite of those of class II. Class IV contained 84 genes that were up-regulated in stage I, but then they rapidly decreased in stage II, and quickly rebounded in stage III. In a previous study [4], transcription factors were found to act as integrators of environmental factors to transmit and amplify adversity signals, regulating the expression of many stress-related genes, and making crops respond to adversity, eventually conferring comprehensive enhanced stress resistance. Therefore, we performed more detailed clustering for the DETs of class IV, which were responding quickly to Pb, and were up-regulated in stage I. The DETs were categorized into 3 clusters according to their expression patterns after stage I (Figure 2C). Among them, TFs in cluster 1 represented genes instantaneously responding to Pb stress, which were induced in stage I, and then returned to an expression level approximate to CK in stages II and III. TFs responding intermittently to Pb treatment were classified into cluster 2, which were up-regulated in stage I and stage III, but showed a lower expression in stage II. The genes in cluster 3 were assumed to be a class of TFs that responded continuously to Pb stress; which were up-regulated at stage I and maintained a higher level through stage III.

### 3.5. Pathways Regulated by DETs under Pb Treatment

To understand more clearly the functional role of the DETs mentioned above, we identified biological pathways enriched with DETs. We found that the DETs were involved in three significant (*p* < 0.05) pathways, including plant hormone signal transduction (17 DETs, 6.07%), basal transcription factors (12 DETs, 4.29%), and base excision repair (2 DETs, 0.71%) (Table 2). Hormones are molecules produced by plant cells in response to environmental stresses and during plant development [20,21]. The plant hormone signal transduction pathway is widely believed to be the most highly relevant pathway in the process of abiotic stress, in which auxin, cytokinin, gibberellin, abscisic acid, ethylene, brassinosteroid, jasmonic acid, and salicylic acid signalling transduction pathways were annotated in the present research.

A total of 17 TFs were detected that participate in the plant hormone signal transduction pathway, where bZIPs were the most significantly enriched with 12 members involved (Table 2). Among them, the orthologues (*AtbZIP20/AHBP-1b/TGA2*, *AtbZIP22/TGA3*, *AtbZIP26/OBF5/TGA5*, *AtbZIP45/TGA6*, *AtbZIP46/PAN*, *AtbZIP47/TGA1* and *AtbZIP57/OBF4/TGA4*) of bZIPs (GRMZM2G132868, GRMZM2G002075, GRMZM2G159134, GRMZM2G161009, GRMZM2G133331, GRMZM2G030877, GRMZM2G125243, GRMZM2G060290, GRMZM2G094352 and GRMZM2G131961) have been proven to play crucial roles in abiotic and biotic stress in *Arabidopsis* [22,23,24,25]. 

### 3.6. Potential Function of bZIP Genes in Pb Tolerance

To reveal the potential relations between bZIP genes and Pb tolerance, we searched the *Arabidopsis* mutant library for orthologues of the 12 maize bZIP genes among T-DNA insertion mutants [26]. We successfully obtained 5 mutants, including tga-1 (*AtbZIP20*, orthologue of *ZmbZIP54*), tga-2 (*AtbZIP47*, orthologue of *ZmbZIP107*), tga-3 (*AtbZIP46*, orthologue of *Zmfea4*), tga-4 (*AtbZIP21*, orthologue of *ZmbZIP79*), and tga-5 (*AtbZIP12*, orthologue of *ZmbZIP102* and *ZmbZIP96*) via the TAIR mutant seed stock [27]. The T-DNA insertions were confirmed to be localized in the exon regions for all the mutants (Figure S3).

To identify the most appropriate concentration of Pb, five concentration levels were set for *Arabidopsis thaliana* wild type (Columbia): Pb(NO_3_)_2_ 50 mg/L; Pb(NO_3_)_2_ 100 mg/L; Pb(NO_3_)_2_ 150 mg/L; Pb(NO_3_)_2_ 200 mg/L and Pb(NO_3_)_2_ 250 mg/L. We found that seedling growth was seriously affected at a concentration of Pb(NO_3_)_2_ 200 mg/L. When it reached 250 mg/L, *Arabidopsis* growth was significantly suppressed. Leaves turned yellow and soon died. Thus, a Pb concentration of Pb(NO_3_)_2_ 200 mg/L was considered a suitable treatment.

**Figure 3:**
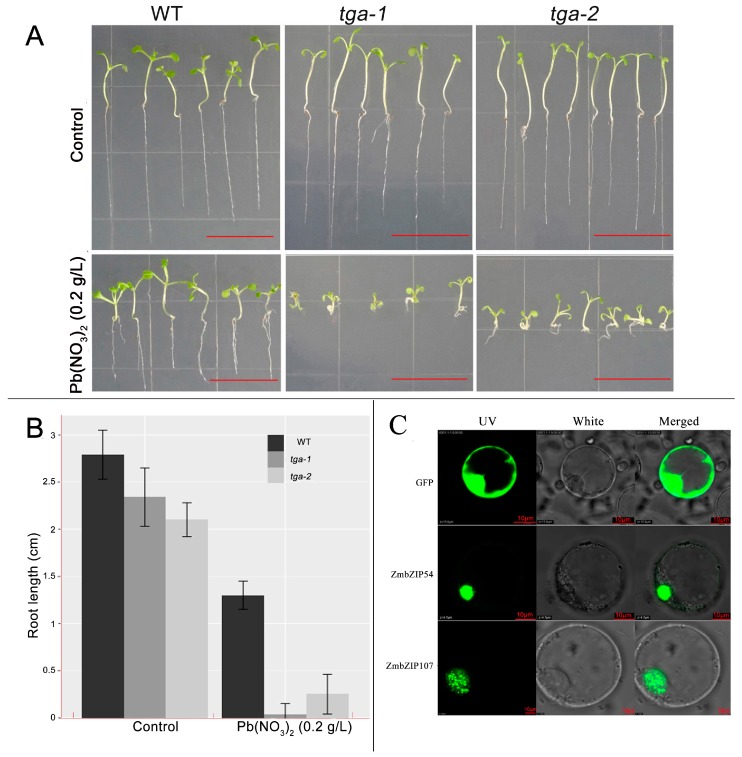
Growth of the *atbzip20* and *atbzip47* mutants under Pb stress and the subcellular localization of *ZmbZIP54* and *ZmbZIP107*. (**A**) Plant growth of wild type and mutants on MS medium with 0 g/L Pb(NO_3_)_2_ and 0.2 g/L Pb(NO_3_)_2_. (**B**) Statistical analysis of root length under control and 0.2 g/L Pb(NO_3_)_2_ stress. (**C**) Subcellular localization of ZmbZIP54 and ZmbZIP107 proteins.

The mutants and WT (Columbia) were then subjected to Pb treatment. The results indicated that plant growth was inhibited by Pb stress for both the wild type and the mutants; whereas only tga-1 and tga-2 showed significantly different responses compared with the wild type. Specifically, there were no differences in plant growth between WT (Columbia) and the tga-1 or tga-2 mutants under control conditions. However, growth inhibition of the mutants was more severe than that in the WT under Pb stress. As shown in Figure 3A, the root length of WT diminished by 53.49% in the Pb treatment compared with that of the CK, while the values of tga-1 and tga-2 reached 98.19% and 88.10%, respectively. The growth of the above-ground parts of the mutants was also clearly inhibited under Pb stress, when compared with WT (Figure 3A,B). These findings demonstrated that knockout of *AtbZIP20* or *AtbZIP47* reduced the Pb tolerance of *Arabidopsis*, but did not significantly influence its growth under normal conditions. As is well known, the bZIP family contains evenly spaced leucine residues, that allow dimerization, and a basic DNA-binding domain [28] that functions in stress signalling in plants. In the previous study, *tag2* showed the strengthened inhibition of root growth under phytoprostane or jasmonic acid relative to the wild type [29], which is similar to the phenotype change of *tga2* under Pb stress in our research. As oxylipins, jasmonates, and phytoprostanes regulate stress responses and diverse physiological and developmental processes [29,30]. Maksymiec et al. reported that heavy metal stress triggered a rapid accumulation of jasmonic acid (JA), which is connected with the mechanism of toxic action of heavy metals in plants [31]. Combining these findings, our results suggest that the transcription factor *AtbZIP47* could play a role in defense response to phytoprostane and jasmonic acid under Pb stress; whereas, mutant *tag1* did not show obvious phenotype changes under phytoprostane nor jasmonic acid when compared with the wild type [29], which is inconsistent with the phenotype change of *tag1* under Pb stress. A possible explanation is that the response of *AtbZIP20* to Pb stress is independent of jasmonates and phytoprostanes. 

### 3.7. ZmbZIP54-GFP and ZmbZIP107-GFP Fusion Proteins Are Both Located in the Nucleus

Plant *bZIP* genes are annotated as DNA-binding transcription factors, and are expected to be present in the nucleus [32]. To verify ZmbZIP54 and ZmbZIP107 protein locations, we subsequently fused *ZmbZIP54* and *ZmbZIP107* with the GFP gene and transformed them into *Oryza* protoplasts by the PEG method [33], respectively. As shown in Figure 3C, fluorescence signals were observed only in the nucleus of the *Oryza* protoplasts transformed with the fusion gene vectors ZmbZIP54-GFP or ZmbZIP107-GFP, but in both the nucleus and the cytoplasm of the *Oryza* protoplasts transformed with the control vector, which showed that ZmbZIP54 and ZmbZIP107 proteins were specifically located in the nucleus.

### 3.8. Disparity in bZIP Expression Levels Accounts for Pb-Tolerance Difference among Maize Lines

**Figure 4:**
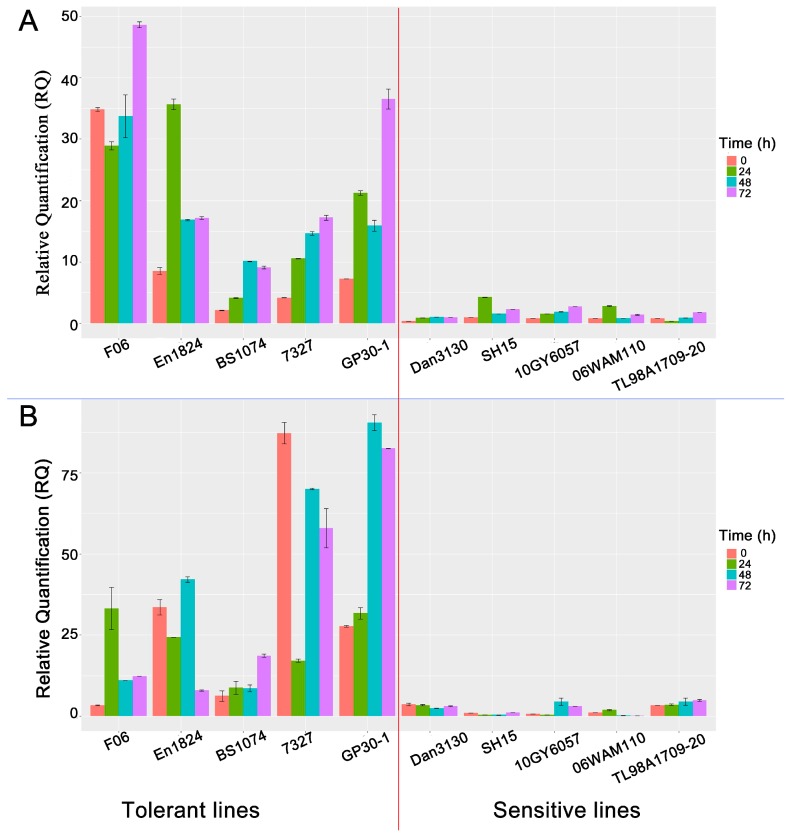
Relative expression levels of the genes *ZmbZIP54* (**A**) and *ZmbZIP107* (**B**) in different lines. Red, green, blue, and purple represent 0, 24, 48, and 72 h after Pb stress, respectively. The expressions of the two genes in line En1824 at 0 h were used as references. The 2^−∆∆Ct^ method was used for calculating relative quantification (RQ).

Differences in Pb tolerance among maize lines have been previously reported [12,34]. To obtain a more detailed molecular mechanism of Pb tolerance in maize, we screened out 5 highly Pb-tolerant inbred lines (F06, En1824, BS1074, 7372, GP30-1) and 5 highly Pb-sensitive inbred lines (Dan3130, SH15, 10GY6057, 06WAM110, TL98A1709-20) from 312 inbred lines, whose Pb tolerance indexes were calculated by the method described by Zhu et al. [35] As shown in Table S7, the Pb tolerance indexes of the sensitive lines ranged from 0.196 to 0.242, and 0.503 to 0.548 for the tolerant lines. First, we amplified and sequenced the coding sequence (CDS) regions of *ZmbZIP54* and *ZmbZIP107* for the 10 lines. Sequence analysis indicated that there are no significant genotype polymorphisms among the various lines (Figure S4A,B). We subsequently conducted qRT-PCR for the two genes in roots of these lines under Pb stress. As a result, *ZmbZIP54* was generally up-regulated in response to Pb stress compared to CK, with the exception of the down-regulated expression at 24 h in lines F06 and 08WSC51 (Figure 4A). In contrast, the expression of *ZmbZIP107* was induced in some tolerant lines but supressed in others during Pb treatment. This also occurred in the sensitive lines (Figure 4B). Remarkably, the expression levels of both *ZmbZIP54* and *ZmbZIP107* were much higher in Pb-tolerant lines than those in Pb-sensitive lines, regardless of CK condition or Pb treatment stages. There was a positive correlation between the expression levels of the two TFs in roots and Pb tolerance for maize inbred lines, which was in accord with phenotypes of the homologous genes, *AtbZIP20* and *AtbZIP47*, in *Arabidopsis*. This suggested that the disparity in *ZmbZIP54* and *ZmbZIP107* expression levels contributes to differences in Pb-tolerance among maize lines. In future research, we will focus on DNA polymorphisms of *ZmbZIP54* and *ZmbZIP107* promoters and their trans-acting factors to gain a comprehensive understanding of the gene expression regulation. In previous studies, tolerance to dehydration and drought in tobacco was increased by over-expression of *PtrABF*, a bZIP TF [36]. Over-expression of the CAbZIP1 gene in *Arabidopsis* enhances disease resistance and tolerance to Methyl Viologen ( MV)-induced oxidative stress [37]. Based on our results, maize tolerance to Pb stress may be improved by over-expression of *ZmbZIP54* and *ZmbZIP107* in roots. 

## Figures and Tables

**Table 1:** The most differentially expressed TFs.

TFs	Gene Identifier	log_2_ (Stage I/CK)	log_2_ (Stage II/CK)	log_2_ (Stage III/CK)
bZIP	GRMZM2G381991	6.38	−6.64	8.33
bZIP115	GRMZM2G157177	8.89	9.32	9.47
bZIP55	GRMZM2G386273	8.18	5.32	7.01
bZIP58	GRMZM2G149040	7.18	8.13	7.59
bZIP71	GRMZM2G019106	−6.64	−6.64	7.88
bZIP83	GRMZM2G000171	7.18	6.64	8.74
CCAAT-binding	GRMZM2G033245	7.18	6.32	−6.64
e2f4	AC233850.1_FG005	7.54	−6.64	5.43
e2f5	GRMZM2G050590	7.18	7.32	6.43
ereb126	GRMZM2G169654	5.36	−6.64	6.74
glk34	GRMZM2G081671	7.18	8.32	8.59
gras11	GRMZM2G097456	−6.64	5.32	7.01
gras13	GRMZM2G140094	−6.64	6.64	6.43
gras14	GRMZM2G070371	−6.64	5.91	7.42
gras38	GRMZM2G098517	7.18	6.32	6.00
gras63	GRMZM2G418899	6.69	5.91	6.43
hb102	GRMZM2G139963	5.36	−6.64	5.43
hb8	GRMZM2G135447	−6.64	−6.64	7.23
jmj5	GRMZM2G070885	−6.64	6.91	−6.64
K-box	GRMZM2G133568	7.69	−6.64	6.43
K-box	GRMZM2G052123	6.95	5.32	5.43
K-box	GRMZM2G033093	6.95	5.32	7.23
K-box	GRMZM2G003514	−6.64	−6.64	−6.64
K-box	GRMZM2G148693	−6.64	−2.01	−6.64
K-box	GRMZM2G137510	−6.64	−5.13	−6.64
K-box	GRMZM2G147716	−2.44	−1.16	−6.64
K-box	GRMZM2G046885	−6.64	0.14	−6.64
K-box	GRMZM2G069370	−6.64	−1.85	−6.64
knox1	GRMZM2G159431	6.95	5.32	7.42
mads41	GRMZM2G018589	8.28	6.64	6.74
obf4	GRMZM2G125243	9.62	9.13	8.81
Pathogenesis	GRMZM2G056729	6.38	6.91	−6.64
TFIIF	GRMZM2G060284	9.37	7.78	8.67
thx23	GRMZM2G156348	−6.64	6.32	8.01
tsh4	GRMZM2G307588	7.18	−6.64	6.00

**Table 2:** DETs annotated in 4 pathways.

Pathway	Gene ID	*Arabidopsis* Homologues	CK (TPM)	Stage I (TPM)	Stage II (TPM)	Stage III (TPM)
DETs annotated in plant basal transcription factors	GRMZM2G017831	AT4G36650	9.52	1.86	11.2	12.22
	GRMZM2G091586	AT2G41630, AT3G10330	18.03	42.84	25.59	31.72
	GRMZM2G045668	AT1G03280, AT4G20340, AT4G20810	2.23	0.41	1.2	1.29
	GRMZM2G064390	AT1G03280, AT4G20340, AT4G20810	36.06	72.43	69.78	82.73
	GRMZM2G114584	AT1G17070, AT2G42330	2.84	11.38	8.2	4.07
	GRMZM2G060284	AT1G75510, AT3G52270	0	6.62	2.2	4.07
	GRMZM2G049091	AT1G75510, AT3G52270	32.62	75.33	33.99	42.44
	GRMZM2G125259	AT1G312240	7.7	2.07	2.4	0.43
	GRMZM2G151717	AT3G02160, AT4G34340, AT5G15570	1.82	1.86	0	0.64
	GRMZM2G173309	AT1G05055	1.01	7.45	6.2	4.93
	GRMZM2G161418	NA	1.01	0	1.8	1.29
	GRMZM2G110076	AT1G54140	3.04	3.31	7.8	4.07
DETs annotated in plant hormone signal transduction	GRMZM2G133331	AT1G68640	3.04	0	1.6	0
	GRMZM2G381991	NA	0	0.83	0	3.22
	GRMZM2G030877	AT1G08320	3.65	8.28	5	3.22
	GRMZM2G132868	NA	4.05	13.24	2.8	3.86
	GRMZM2G125243	AT3G12250, AT5G06950, AT5G06960	0	7.86	5.6	4.5
	GRMZM2G002075	AT1G45249, AT1G49720, AT3G19290, AT4G34000, AT5G42910	5.06	6	11.4	7.72
	GRMZM2G159134	AT2G41070, AT3G56850	2.84	2.07	5.2	10.29
	GRMZM2G161009	AT2G41070, AT3G56850	5.67	16.97	12.4	12
	GRMZM2G060290	AT5G06839	3.85	36.63	8.8	21.01
	GRMZM2G361847	AT3G12250, AT5G06950, AT5G06960	61.59	81.75	117.76	147.25
	GRMZM2G094352	AT1G22070, AT1G77920, AT5G10030, AT5G65210	98.05	168.04	115.16	215.19
	GRMZM2G131961	AT1G22070, AT1G77920, AT5G10030, AT5G65210	113.05	95.4	77.37	237.7
	GRMZM2G024973	AT1G14920, AT1G66350, AT2G01570, AT3G03450, AT5G17490	160.05	63.74	42.79	55.08
	GRMZM2G330012	AT3G20220	1.82	1.03	4	2.79
	GRMZM2G049229	AT1G32640, AT4G17880, AT5G46760, AT5G46830	134.52	125.2	56.38	117.67
	GRMZM2G001930	AT1G32640, AT4G17880, AT5G46760, AT5G46830	528.97	287.87	149.55	230.84
	GRMZM2G030280	AT3G12250, AT5G06950, AT5G06960	13.98	31.46	11.4	23.15
DETs annotated in plant base excision repair	GRMZM2G012654	AT2G27470	7.09	14.49	12.4	10.72
	GRMZM2G139031	AT1G21710	2.03	1.24	5.8	2.36

## Supplementary Materials

The following are available online at www.mdpi.com/2073-4425/8/9/231/s1. Figure S1. The carrier of subcellular localization. Construction of the expressive vector by the restriction endonuclease BamHI. Figure S2. Pie charts show different TF families present in the TF libraries. “Low expression TF family” represents the TF families which including only one or two members. “Unknown TFs” represent TFs that cannot be classified into any known family. Figure S3. Insertion sites of *Arabidopsis* mutants. Exon sequences are represented by blue rectangles, and light blue rectangle designate UTR regions. Introns are indicated by black lines. T-DNA insertions are represented by inverted triangles. Figure S4. Comparison of the amino acid sequences of *ZmbZIP54* and *ZmbZIP107* in different lines. Table S1. Formula of nutrient solution for culturing maize seedlings. Table S2. Real-time PCR primers of differentially expressed TFs. Table S3. Differentially expressed TFs. Table S4. TF numbers of every differentially expressed TF family in 3 stages. Table S5. Relative expression levels of the randomly selected DETs at different development stages without Pb treatment (compared with 0 h). Table S6. TFs in 4 classes. Table S7. Pb-tolerance indexes for the 10 inbred lines in this study.
